# The Effect of Adult Aggression on Habitat Selection by Settlers of Two Coral-Dwelling Damselfishes

**DOI:** 10.1371/journal.pone.0005511

**Published:** 2009-05-13

**Authors:** Ofer Ben-Tzvi, Moshe Kiflawi, Omer Polak, Avigdor Abelson

**Affiliations:** 1 The Interuniversity Institute for Marine Sciences of Eilat, Eilat, Israel; 2 Department of Zoology, George S. Wise Faculty of Life Sciences, Tel-Aviv University, Tel-Aviv, Israel; 3 Department of Life Sciences, Ben-Gurion University, Be'er Sheva, Israel; Lund University, Sweden

## Abstract

Coral-reef fishes experience a major challenge when facing settlement in a multi-threat environment, within which, using settlement cues, they need to select a suitable site. Studies in laboratories and artificial setups have shown that the presence of conspecific adults often serves as a positive settlement cue, whose value is explained by the increased survival of juveniles in an already proven fit environment. However, settlement in already inhabited corals may expose the recruits to adult aggression. Daily observations and manipulation experiments were used in the present study, which was conducted in the natural reef. We revealed differential strategies of settlers, which do not necessarily join conspecific adults. *Dascyllus aruanus* prefer to settle near (not with) their aggressive adults, and to join them only after gaining in size; whereas *Dascyllus marginatus* settlers in densely populated reefs settle independently of their adult distribution. Our results present different solutions to the challenges faced by fish recruits while selecting their microhabitat, and emphasize the complexity of habitat selection by the naïve settlers. Although laboratory experiments are important to the understanding of fish habitat selection, further studies in natural habitats are essential in order to elucidate the actual patterns of settlement and habitat selection, which are crucial for the survival of coral-reef fish populations.

## Introduction

The vast majority of reef fishes (as well as many other coral-reef organisms) have a bipartite life cycle divided between a pelagic larval stage and a benthic adult stage. A critical process of the transition between the two stages is that of settlement in the reef. In addition to recruitment intensity and timing, replenishing of existing fish populations and colonizing new habitats is conspicuously affected by factors acting during or immediately after settlement, such as competition [1], predation [2] and habitat selection [3], [4]. Following settlement and recruitment, most fishes remain at the same location, whether an entire reef patch or just part of a single coral head [5], throughout their entire lives.

Accomplishment of a successful recruitment necessitates identification of the appropriate reef patch and a suitable microhabitat within this patch. Locating such a reef from within the boundless oceanic matrix is probably one of the greatest challenges that reef fishes face, and requires highly developed sensory capabilities [6], [7]. After locating the reef, the next stage is that of a particular microhabitat selection from within the selected patch. Cues used for microhabitat selection or rejection must involve factors directly affecting the fitness of the fish [8], which may use many such cues in order to locate the best available microhabitat (within a given reef patch) for their requirement. A number of studies have examined the selection of specific microhabitats and the cues used for habitat selection by recruiting reef fishes [9]–[17]. However, only a few works have examined the settlers' choices in natural reef patches, where the settling larvae need to select their microhabitat from a multi-option, complex environment during the (usually moonless) night [18].

One of the decisions that the fish have to take when selecting their microhabitat is that of whether or not to join an existing school of conspecific adults. There are several immediate costs and benefits to a settler in joining conspecific adults. Some of these are also long-term and appear later, when the juvenile is part of a group. While the presence of conspecific adults might be an indicator of a suitable habitat, this will may not always be so for the small settlers, due to their need for an habitat that is different in its complexity and density from the adult habitat. For example, recruits of pomacentrids avoid some of the more open corals [19] possibly in order to reduce the risk of predation by small predators able to maneuver in between less dense coral colonies [20]. Moreover, adults may increase the risk of predation for the juveniles living with them since they often push them to the edges of the shelter [20]–[22]. As a result, the presence of adult pomacentrids sometimes reduces the recruitment of conspecific juveniles [21], [23], [24]. On the other hand, there are many reports that aggregations and the presence of adults reduce the risk of predation and increase the survivorship of juveniles within such an aggregation [25], [26] through better vigilance by more fish in a larger foraging space [27], [28], and the aggressive behavior of adults towards potential predators [29]. Moreover, by joining conspecifics the young recruits learn faster predator recognition from the adults [30]. The enhanced safety allows the juveniles more foraging time [31] within a larger space [32] and higher up in the water column, where more prey are expected to be found [33]. The better foraging conditions, however do not necessarily mean higher food uptake by the smaller fish. On the contrary, aggregated young fish eat less [34]–[36], and reach smaller prey and of inferior quality than those they can obtain in the absence of their larger conspecifics [37].

Some planktivorous coral-dwelling damselfishes have been shown to prefer to recruit to corals hosting conspecific adults, using a chemical cue (among other cues) to identify these corals [11], [12], [14]. These findings were obtained by studies that examined the abilities and choices of the recruiting fish in the laboratory and/or in an artificial set-up in the sea. However, it is possible that the presence of adults of some species, such as those of *Dascyllus aruanus* (Linnaeus, 1758), might prevent recruits of other species from settling in the same habitat [16], [38].

In the present study we examined, in a natural coral reef in Eilat (Gulf of Aqaba, Red-Sea) habitat selection by recruits and the relationship between adults and recruits of two damselfishes, *Dascyllus marginatus* (Rüppell, 1829) and *D. aruanus*, which share the same habitat. Our aims were: a) to study the habitat selection of settlers regarding the presence of conspecific adults by examining whether their reported choice of colonies inhabited by conspecific adults [16], [39] also occurs in the natural reef, where the fish have to react to many cues and threats; and b), the effect of adult-settler relationships on the settlers' decision, focusing on whether and how the conspecific adult behavior (and mainly *D. aruanus* aggression) affects this decision. Damselfishes tend to be territorial and quite tenacious and aggressive defenders of their spot on the reef, which make them a good model for this study's questions, due to the expected conflict between the value of conspecifics as a cue for a suitable habitat and the cost of settling near aggressive conspecifics.

## Materials and Methods

The experimental manipulations with fish in this study were conducted according to the Israeli guidelines for animal welfare and with the permission of Israel's Nature and Parks Authority. Maximum effort was made to safely return the juvenile fish used in these manipulations to their original habitat.

### The studied species

We investigated the settlement patterns of two planktivorous coral-dwelling pomacentrids (damselfishes), *D. marginatus* and *D. aruanus*. Both are common at the study sites, at which they were observed to settle from June to December 2004–2007. As many other pomacentrides, the settlers of these fishes resemble the adults in appearance [40], and settle into some of the same species of corals as inhabited by their conspecific adults [19]. Since they settle after metamorphosis (i.e. their colors and morphology resemble those of adults) and, in many cases, directly within a habitat similar to that of the adults, they are usually considered as recruits from the time of settlement (unlike settlers that hide in a temporary habitat where they undergo a post-settlement morphological transition, after which they recruit to their final habitat) [40] (however, we use the term ‘settler’ since, at least for *D. aruanus*, a difference between settlement and recruitment was found). *D. marginatus* can be found in many branching coral species, mostly *Acropora* spp and *Stylophora pistillata* [41], from near the coastline (depth of about 2 m; almost at the coastline) down to more than 40 m depth. They are organized in stable territorial harems in which one or a few males dominate the females [42]. Harem sizes in the study area usually varied from two to ten fish, but there are also some ‘bachelors’ (solitary individuals of which at least some were male as identified by their up and down mating dance [41] and their guarding of nests) occupying coral colonies by themselves, and, on the other hand, some bigger groups of >30 fish. The bigger harems are usually found in the bigger *Acropora* colonies. *D. aruanus* is common throughout the reefs of the Indo-Pacific and has been extensively studied, but is less abundant than *D. marginatus* in our study area, where it occupies the same coral species as *D. marginatus* [41]. The biology and ecology of both fishes are similar [41], [43], [44]. There is a clear social ranking of the fish within their harems [37], [41], [44]. The fishes are all female when they recruit, with some of them changing sex to male upon obtaining a high rank [44]. There is a difference in the dispersal depths of the two species at the study area, with *D. aruanus* rarely being found deeper than 15 m. It is abundant in the lagoon and areas protected from currents and waves, where *D. marginatus* is almost absent. The sizes of *D. aruanus* harems in the study site resemble those of *D. marginatus*. At depths where both species are found, they may co-inhabit the same coral colony [41]. Different mixed species groups vary in the numbers of individuals of each species, and the majority can be of either species. The settlers of both fish species are observed mainly in dense *Acropora* spp. coral colonies (Ben-Tzvi unpublished data).

### Settlers' distribution data collection

The present study took place in the coral reefs of Eilat at the northern tip of the Gulf of Aqaba (∼29°30′N 34°55′E). We monitored by means of daily censuses the recruitment to two reef-patches, one opposite the Interuniversity Laboratory (IUI) and the other within Eilat's Marine Nature Reserve. These patches are several hundred m long, occupying a belt with a varied width of 30–60 m between the depths of 2–10 m. At each site, tagged branching corals were monitored along a fixed 2 m wide zigzag belt transect with 4 legs of about 50 m each. The tagged corals were of the same species and size (diameter >10 cm) that both fish species are known to inhabit and to select for settlement. All suitable corals within the transect were tagged. We recorded the null state of each tagged coral before the beginning of the recruitment season. This included the coral species and the number of adults of the two *Dascyllus* species inhabiting it (if there were any). The recruitment to Eilat's coral reefs is characterized by recruitment events of different durations and intervals (also of different durations) with no recruitment between them [22], [45]. In 2004 we recorded four such events. However in the two first events recruitment was very weak and we observed only a few *D. marginatus* and no *D. aruanus* settlers. In the other two events, one of four weeks during August–September 2004 and the second of five weeks during October–November 2004, we observed intensive recruitment, including the two study species [22], [45]. The data from these two major events were used for this study. The null state regarding the number of adults was similar in both recruitment events but at the beginning of the second event many corals were inhabited by new settlers (including colonies which had been empty before). A total of 398 coral colonies were tagged. We counted the settlers at each tagged colony daily throughout the entire recruitment season and recorded their distribution in the corals.

### Settling data analysis

The tagged corals were divided into four categories: colonies hosting adult *D. marginatus*, corals hosting adult *D. aruanus*, colonies hosting both species, and colonies with no adults of either species (Table 1). We used a chi-square test to compare the observed number of settlers in each coral category and the expected number according to the relative abundance of colonies of each category. We conducted the test twice: first the distribution of actual numbers of all settlers in the tagged corals, and second, the binary data (present/absent), which ignore the number of fish per coral (the number of fish settled into each tagged colony as well as the different colony sizes were not taken into account in these comparisons). The first comparison reveals the category that the fish had chosen and the second shows the proportion of chosen corals from each category. Theoretically, these two distributions can differ. For example: if many fish choose only a few corals from a particular coral category, the relative number of settlers into these corals can be high while the relative selected coral is low. We performed the comparisons twice for each of the two major recruitment events.

**Table 1:** Distribution of the tagged corals according to their host status (populated by *D. aruanus*, *D. marginatus*, both, or unpopulated).

	All tagged corals	*S. pistillata* excluded
*D. aruanus*	38	36
*D. marginatus*	176	115
Both species	9	8
Uninhabited	175	126
Total	398	285

The right column shows the distribution of colonies when *S. pistillata* colonies are excluded.

The distribution of settlers might be affected by the presence of conspecific juveniles that had settled a few days earlier [12], [22]. It is not known, however, whether the juveniles of the studied species affect the recruitment of new conspecifics, and if so, in what way. If such an effect does exist, it may induce a biased distribution (either increase or decrease settlement to the coral they inhabit) of the later settlers. This was an additional reason why a present/absent analysis was also employed. We compared the number of coral colonies of each of the above-mentioned categories that received settlers to the expected number (the proportion from the entire sample multiplied by the number of colonies in the category).

### Post settlement migration

We also observed and recorded movements of juvenile fish (after settlement) between the tagged corals. Since fish grow fast, an experienced observer can distinguish between those that have just settled and those that are two weeks or more post-settlement (the age and size of both studied species in settlement is very similar) [45]. The fish themselves were not tagged and, therefore, it was impossible to identify which fish had migrated from/to which coral. In spite of this, it was possible to identify and document appearances/disappearances in/from the tagged corals of juvenile fish (which had settled a few weeks earlier and which, due to their size, are easily distinguished from the settlers).

### Supporting qualitative data

We obtained qualitative data on the settlement and juvenile migration and on the relationship between adults and juveniles of the two species by field observations, using SCUBA. These data were collected from the entire reef patch areas (from 10 m depth to the shallowest corals in the transacts' area), including the narrow (10–30 m wide) and shallow (<3 m depth) lagoon of the Marine Nature Reserve, where *D. aruanus* is the only abundant coral-dwelling damselfish. These data were collected about once a week in addition to the data obtained from the tagged corals. We have searched the reef in order to identify settlement. These were considered important complementary data, especially in the lagoon where settlement differed from other areas (see Results and Discussion) Based on these observations, we made an attempt to determine the foraging positions of the juveniles in the presence or absence of adults. We also obtained (by observation) data on how adults and juveniles share the space in the hosting corals and their surroundings. We collected these data from a distance of 3–4 m from the coral. From this distance the fish are not disturbed and they continue to forage normally. These supporting qualitative data are important in the interpretation of the obtained quantitative data, regarding the costs and benefits to settlers of joining an existing group of conspecific adults, and the implications of adult behavior for the balance of these costs and benefits.

### Distances from D. aruanus adults

The distance between the selected coral and the nearest coral with conspecific adults can also be indicative of the settlers' preferences. We measured these distances to the nearest colony inhabited by *D. aruanus* for both for corals that received conspecific settlers and for all the tagged suitable corals (*Acropora* spp. of the species and size that settlers may choose). We did this in part of the transect at Eilat's Marine Reserve located in the area in which most *D. aruanus* settlers selected. We measured the distance from the edge of each coral to the edge of the nearest coral hosting adult *D. aruanus*, whether a tagged coral or not. The distance distribution of all suitable corals in the chosen part of the transect was compared to that of the settlers' recipient corals. We applied Kolmogorov-Smirnov test in order to determine whether the distribution of distances of the selected corals differed from that of all suitable corals.

### Adult aggression toward juveniles

To determine whether different levels of adult aggression on juveniles might affect the settlers' habitat selection, we introduced juvenile fish of both species into corals hosting adults of these two species (one species at a time introduced into a coral hosting one of the species adults only). The experiment took place in an area in which divers are very common and the adult fish are used to them. The fish retreat to their host coral when the divers approach and hide there until the divers leave, at which time the fish immediately come out and continue foraging. To ensure that the effect of stress on the adults from our presence did not prevent them from reacting to the juveniles, it was decided to continue each individual observation either until there was a reaction or the adults resumed foraging.

We collected juvenile fish of the smallest size (presumably not more than 1 week on the reef) from their corals. To collect the fish we sprayed clove oil under their host coral. In such situations some of the inhabiting fish leave the coral (and others, mainly adults, do not). We collected juveniles that had left the coral near the coral edges, using hand nets (others, which were not collected, returned to the coral within a short time since the clove oil rose upwards quickly). A diver then immediately released the collected juveniles individually a few cm (≈10 cm) from another coral that was inhabited by adults of one of the two species only. After releasing the fish, the diver withdrew to a distance of ∼2 m to observe the young fish entering among the coral branches and the reaction of the adults to it (a diver at this distance does not prevent the fish from foraging normally). Since the juvenile fish was released close to a specific coral, its first choice was to escape into this coral. At this stage the diver was quite close and the inhabiting adults were hiding in between the coral branches. This situation resulted in an immediate encounter between the introduced settler and the inhabiting adults. However, the released fish were either able to escape from this coral (as happened many times; see Results) or to avoid entering at all (four such cases occurred and the fish were recollected and returned to their original coral; these cases have been excluded from the statistics). At the end of each experiment the young fish were recaptured and released back to their original coral of capture (except for four fish that were preyed upon; see Results). We planned to recapture 30 seconds after release, but in most cases the experiment was terminated much earlier.

We defined four categories of adult reaction to the encounter with the introduced juvenile: 1. indifference, all adults ignored the new juvenile and left the coral to continue foraging; 2. attack, at least one adult attacked the juvenile within the coral but let it stay there; 3. attack and expulsion, at least one adult attacked the juvenile and expelled it while chasing it several meters from the colony (in these cases we intervened and recaptured the juvenile); and 4. predation, an adult swallowed the juvenile. As far as we could observe, the attacks (categories 2 & 3) comprised a brief chase with no physical contact or injury. We documented only the type of reaction that we considered to contribute the most important data. Moreover, the short time of the reaction make it almost impossible to measure its duration. Moreover, the measurable time from release to the initiation of the reaction could also be affected by factors like our presence and the time it takes for the adult to identify the introduced fish (and thus not comparable). We assumed that the juveniles were highly stressed and, therefore, that the data on their behavior were without value. Therefore, we did not collect these data.

Since some of the counts of this experiment were zero or very low, chi-square and G test were not appropriate (the null distribution of the statistic deviates from the expected). Thus, we used the two-sample Kolmagorov-Smirnov test to compare the behavioral response of adult *D. marginatus* and *D. aruanus* to the presence of new recruits of either species. Owing to zero or low counts in some of the response categories, statistical significance was evaluated by bootstrapping. Briefly, the null probability of sampling each behavioral response was calculated as the total number of counts in that response category (i.e. summed across the two ‘treatments’ being compared), divided by the grand total number of counts (i.e. summed across the four response categories of both treatments). These probabilities were then used to randomly distribute the observed number of counts, per treatment, across the four response categories. The procedure was repeated to generate 5000 such paired bootstrap samples, from which we generated the null distribution of the KS statistic. Rejection of the null hypothesis, that responses to paired treatments were drawn from the same population, was based on observed values of the statistic exceeding the 95th percentile of the null distribution.

## Results

### Settler distribution

Although there were some differences in the distribution of the fish settlers between the inhabited and uninhabited corals in the two recruitment events (Table 2), there was no significant settlement with conspecific adults at any event for both the studied species. The number of settlers of both species in corals with adult *D. aruanus* was lower than that randomly expected (Table 2), but χ^2^ tests showed no significant differences between the randomly expected and the observed distributions of the settlers of both fish during both recruitment events (raw data; Table 2) The number of settlers observed in *S. pistillata* colonies was negligible (e.g. 2 out of 753 *D. marginatus* in the first recruitment event, where *S. pistillata* comprised ∼35% of the tagged colonies). It was also much lower than might have been expected based on the relative abundance of inhabited colonies of this species in the study area (∼60% of the tagged *S. pistillata* were inhabited by *Dascyllus* spp). Relatively large specimens of *Pseudochromis olivaceus* that inhabit these corals were observed preying on settlers of *Dascyllus* spp (predation was observed occasionally, but with no quantitative data). It should be noted that settling fish were observed in <10 cm diameter colonies that were not tagged and not inhabited by *P. olivaceus*. To examine the effect of the *S. pistillata* colonies (in most of which, as noted, settlers were never observed) on the results, we also calculated the expected distributions when these corals were excluded (raw data; Table 2). No correlation was found between the number of adults and number of settlers in the same coral (regression; for each species in each event p>0.7 and r^2^<0.13).

**Table 2:** Comparison of the distribution of settlers of *D. marginatus* and *D. aruanus* among corals with and without conspecific adults presented as the percent of settlers observed on each coral category.

Fish sp. & settlement event	Corals with *Dm*	Corals with *Da*	Corals with Both species	Uninhabited corals	χ^2^	n
*Dm* Aug–Sep	70.65	1.73	0.66	26.96	1.677	753
*Da* Aug–Sep	37.50	5.00	2.50	55.00	0.324	40
*Dm* Oct–Nov	43.14	2.94	1.96	51.96	0.53	204
*Da* Oct–Nov	36.73	6.12	2.04	55.10	0.231	49
Expected	44.22	9.55	2.26	43.97		
Ex. *S.p.* excluded	40.35	12.63	2.81	44.21		

Values are based on the data which were documented in the corals of each category during an entire recruitment event from all settlers of the species at the same event. The distribution of each fish species is represented separately for each recruitment event. The expected random distribution is actually the percentage of coral colonies of each category from all tagged colonies. The expected distribution when all *S. pistillata* are excluded is also provided. n = the total number of settlers of each species per event. The results of χ^2^ test comparing the observed and expected distribution of selected corals is provided for each species at each recruitment event among all tagged corals (critical χ^2^ = 7.815; α 0.05) *Dm* = *D. marginatus*; *Da* = *D. aruanus*; *Sp* = *S. pistillata*.

The presence of con- and hetero-specifics did not influence the settlement rate in either *Dascyllus* species. Juveniles settled to inhabited and uninhabited corals in proportion to their abundance (present/absent; Table 3). The distribution was not significantly different from the distribution of the corals in the different categories (χ^2^ test; Table 3). It should be noted, however, that about half of the tagged corals (152 out of 285; *S. pistillata* excluded) were selected by *D. marginatus* settlers in the first recruitment event. 87% of these colonies were selected again in the second event. Only two colonies that were selected by *D. marginatus* in the second recruitment event had not been selected in the first event. Thus, in the second event practically all the selected colonies were inhabited (some of them only by juveniles). Such a possible effect of the presence of juveniles was minor for *D. aruanus*, in which only 12% of the colonies that were selected by this fish were selected in both events.

**Table 3:** Comparison of the percentage of selected corals from each coral category out of the entire selected corals, compared with the expected random percentage (i.e. the percentage of corals of each category out of all the tagged corals).

Fish sp. & settlement event	Corals with *Dm*	Corals with *Da*	Corals with Both species	Uninhabited corals	χ^2^	n
*Dm* Aug–Sep	56.58	4.61	1.97	36.84	0.389	152
*Da* Aug–Sep	42.86	3.57	3.57	50.00	0.747	28
*Dm* Oct–Nov	39.81	3.88	2.91	53.40	0.47	134
*Da* Oct–Nov	38.46	5.13	2.56	53.85	0.3	39
Expected	44.22	9.55	2.26	43.97		

Data are presented separately for each fish species (*Dm* = *D. marginatus*; *Da* = *D. aruanus*) at each recruitment event. n = number of colonies selected by the species in the recruitment event. The results of χ^2^ test comparing the observed and expected distribution is provided for each species at each recruitment event among all tagged corals (critical χ^2^ = 7.815; α = 0.05).

### Spatial distribution of *D. aruanus* in relation to adult conspecifics

**Figure 1:**
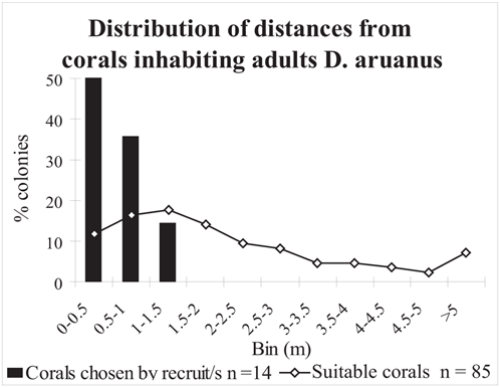
The distribution of distances of tagged corals in part of the transect from corals hosting *D. aruanus* adults. Columns: corals that were chosen by *D. aruanus* settlers. Line: all suitable corals (species and size that *D. aruanus* settlers usually choose).

The distribution of distances of corals selected by *D. aruanus* settlers from corals hosting conspecific adults was found to be non random (Fig. 1; Kolmogorov-Smirnov test p<0.001, Z = 0.74). 50% of the settlers chose colonies that were 50 cm or less from inhabited colonies and all the settlers settled less than 150 cm from conspecific adults. Only 46% of the potentially suitable colonies were less than 150 cm from a colony inhabited by adult *D. aruanus* and about 10% of them were more than 5 m from the closest inhabited colony (Fig. 1).

In mid-October 2004, about six weeks after termination of the first recruitment event, 27 young *D. aruanus* fish were still to be found in the corals they had selected during their settlement (all these corals were of the two *Acropora* species favored by settlers of these fish). In the following three weeks 11 of these fish disappeared (from both *Acropora* species). Concurrently, eight juvenile *D. aruanus* of the same size (age) settled in corals with conspecific adults in which no direct settlement (of naïve fish) had been previously observed. Moreover, at the same time more than 30 young *D. aruanus* at about two months post-settlement stage, settled in a coral patch in the lagoon of Eilat's Marine Nature Reserve. This patch had been surveyed several times between August and mid-October and no *D. araunus* settlers had been observed there. When the young fish did appear, they were found only in corals hosting conspecific adults. It should be noted that, in contrast to 2004, in summer 2005 many *D. aruanus* did settle directly in the lagoon. As observed in 2004 in the outer reef, in the lagoon in 2005 too all the observed recruits selected their habitat in uninhabited colonies (mostly *Seriatopora caliendrum*) close to colonies (usually *Acropora* spp) inhabited by conspecific adults and not with the adults. No quantitative data were collected for this year.

### Adult aggression toward juveniles

The results of the aggression experiment are presented in table 4. Kolmogorov-Smirnov test with bootstrapping reveals that Adult *D. aruanus* responded differentially to conspecific and congeneric juveniles (p = 0.019), showing higher aggressiveness towards the latter. On the other hand, the response of adult *D. marginatus* to conspecific and congeneric juveniles was indistinguishable (p = 0.866); and, overall, significantly different from the response of *D. aruanus* to juveniles of either species (p<0.001). In general, adult *D. aruanus* tended to be more aggressive in their response to juveniles than adult *D. marginatus* (Table 5).

**Table 4:** The reaction of *D. marginatus* and *D. aruanus* adults to juveniles (a few days post-settlement) of both species that were introduced into their coral.

Adult	Juvenile	Indifference	Attack	Attack & expulsion	Predation	Total
*D. marginatus*	*D. marginatus*	7	5	4	-	16
*D. marginatus*	*D. aruanus*	7	8	4	-	19
*D. aruanus*	*D. marginatus*	-	-	12	4	16
*D. aruanus*	*D. aruanus*	-	6	13	-	19

One juvenile of one species at a time was released into the coral and the reaction of the inhabiting adults (always of only one species) was classified as one of four categories.

**Table 5:** Results of two-sample Kolmogorov-Smirnov test comparing the reaction adults towards juveniles.

Reaction 1: adult/juvenile	Reaction 2: adult/juvenile	K-S Z value	p
*D. aruanus*/*D. marginatus*	*D. aruanus*/*D. aruanus*	0.316	0.019
*D. marginatus*/*D. marginatus*	*D. marginatus*/*D. aruanus*	0.069	0.866
*D. marginatus*/both species	*D. aruanus*/*D. aruanus*	0.456	<0.001
*D. marginatus*/both species	*D. aruanus*/*D. marginatus*	0.771	<0.001

Results are presented as Z values. Z values were compared to the distribution of 5000 such paired bootstrap samples to find the probability to obtain this value randomly (presents as p).

## Discussion

The continuous existence of the harem, the typical social structure of both *D. marginatus* and *D. aruanus* [44], depends on the supply of young female recruits to the existing schools. The males, the highest ranking fish of the harem, benefit from new females joining the harem. There is no cost for them from the joining of young fish since they forage farther upstream and higher in the water column and occupy the best shelter. Thus they face no real competition for food supply or shelter. Settling fish may gain some advantages by selecting a coral colony that hosts conspecific adults, including enhanced survivorship (due to better protection from predation) [25], [26], and settling in a coral that has already proven its suitability for the species (although an empty coral does not necessarily means that it is less suitable, it was shown that the fish do discriminate between corals due to qualities which are unknown to us [22]), and which also has easy access to potential future mates. Since both species are very similar in their needs (food and shelter), behavior and threats, settling with heterospecifics might have similar costs and benefits, with two exceptions: the aggressiveness of *D. aruanus* adults towards other species [15], [46] and easy access to potential future mates. However, contrary to our expectations [16], [45], the settlers' distribution reveals that this was not the case with our studied species. According to our data, no preference was shown for a given situation of a coral (with or without adults) during microhabitat selection; and, thus, it may be concluded that the habitat selection by settlers of both species is random with respect to the presence of adults.

The repeated selection of the same coral colonies in the two recruitment events suggests that, at least for *D. margintus*, the coral selection (regardless of conspecific adults' presence) is not random. If this is the case, the expected pattern of preferred settlement with conspecific adults and the avoidance of other adults should, therefore, be rejected for both studied species. However, this similar settler distribution suggests that the presence of conspecific juveniles might serve as a recruitment cue, as shown for another pomacentrid, *Chromis viridis* [12]. It should be noted, however, that the effect of the presence of the studied species' juveniles (from the first event) on the microhabitat selection by their settling conspecifics during the second event is not clear. On the one hand it can be argued that it was the presence of these juveniles in most of the chosen corals (including those without adults) in the second event that resulted in the similarity between coral selection in both events. If this is so, at least at the second event the distribution of settlers does not seem not to be random regarding the presence of conspecific juveniles. On the other hand, it was shown that *C. viridis* also select the same coral colonies also when there are no conspecifics (adults or juveniles) probably because of different (unknown) qualities of the corals [22]. We can not exclude that it is the same phenomenon that caused the similar selection here. If this is the case, the random distribution regarding conspecifics (both adults and juveniles) remained the same in the second event. Revealing whether the studied species' juveniles affect their conspecific settlers' habitat selection requires farther research. Since this was not known we concentrated here on the distribution of settlers regarding their conspecific adults and this seems to have beene random also in the second event.

Nevertheless, three separate observations suggest that priorities in habitat selection do exist to some extent (especially for *D. aruanus*): 1) the negligible number of settlers that were observed in *S. pistillata*; 2) the relatively low number of settlers in corals with *D. aruanus* adults (although not significant for *D. aruanus* it contrasts with expectations); and 3) the closeness of the colonies that were selected by *D. aruanus* settlers to corals hosting their conspecific adults. Regarding the first two observations, it is not known whether they are the result of habitat selection or of other processes. The settling fish may have avoided such relatively open corals as *S. pistillata* [19], corals in which a predator (e.g. large *P. olivaceus* fish in *S. pistillata*) was present, or they may not have been observed there because they had succumbed to predation upon at arrival. Similarly, only a few fish may have chosen to settle in corals hosting the aggressive *D. aruanus*; or, they were not found there because they had already been expelled by the coral's inhabitants.

The third observation (which applies only to *D. aruanus*), however, is in our opinion different, since in this case the settlers were present and had evidently preferred to settle near their conspecific adults. The observations from the shallow lagoon in 2005 support the quantitative data from 2004, showing that this pattern is consistent and independent of differences in recruitment intensities and sites. It is known that *D. aruanus* display aggressive behavior towards competitors, including conspecifics [46] and potential predators [47], as well as against settlers of other species [14]. Two observed phenomena indicate that the few settlers in corals inhabited by adult *D. aruanus* might have been stressed by the inhabitants' aggressiveness. First, their foraging behavior differed from that of the other settlers. Instead of foraging in front of their host colony on its upstream side as expected [48] and as most settlers were observed, they were observed foraging on the downstream side, usually at the coral edge. Second, these settlers' coloration indicated that they were stressed (i.e. the front half of *D. marginatus* becomes darker when they are stressed while in *D. aruanus* the white lines become grey). We rarely observed other settlers with such coloration. Based on these, we hypothesized that the avoidance by most settlers of joining *D. aruanus* adults occurred due to the adults' aggressiveness towards settlers, including their conspecifics. Our experiment was designed to examine this hypothesis.

This experiment was not designed, however, to simulate habitat selection during settlement since settlement occurs at night and settlers are naïve (and we used non-naïve fish during the day). The experiment examined the differences in adults' behavior toward juveniles. The results have clearly demonstrated that *D. aruanus* juveniles too are subjected to aggression by conspecific adults, and thus strengthen our hypothesis that adult aggression explains (at least partially) the observed habitat selection by settling *D. aruanus*. The aggressive behavior of *D. aruanus* adults places the young settlers in a dilemma: whether to choose corals hosting their conspecific adults and gain all the benefits from joining an existing adult group; or to avoid the adults' aggressive behavior and choose non-occupied corals, which may put them at higher risk. As shown in many previous studies [27], [28], [47], settlers prefer to gain the benefits of better protection, and pay the cost in slower growth and even delayed sexual maturity [19]. The aggressiveness of adult *D. aruanus* seems to make this cost too expensive, however, as represented by the few settlers that had chosen corals with resident *D. aruanus* adults and were forced to forage in the least favorable position, the downstream edges of the coral. The initial settlement near adults, but in colonies not hosting adults, followed by a later migration to corals with adults (after some gain in size), is a possible low-cost solution to such a dilemma. During the first weeks following settlement the fish may gain some benefit by partially staying under the adults' “umbrella” without paying the full price. However, as shown by the influx of juveniles into the lagoon, the young fish do not necessarily migrate to their new habitat by joining the closest inhabited coral. The migration to and joining of an existing school should be considered as the recruitment of these juveniles (settlement and recruitment are usually not distinguishable for pomacentrids [40]). Whether the fish recruit to an adjacent occupied colony or to a more distant one, replenishment of the existing harems is achieved.

Although the level of aggression exhibited by adult *D. marginatus* was much lower than that displayed by *D. aruanus*, we found no significant preference of the settlers of the former to settle with their conspecific adults. The presence of conspecific adults did not seem to be a consideration for these settlers when they selected their microhabitat at the study site. No juveniles from the year of study were observed in many otherwise occupied coral colonies (the majority of them *S. pistillata*). This observation could be hard to explain if the balance between costs and benefits had changed towards more beneficial settlement with adults due to the lack of aggression. However, it seems that the benefits' side too is concurrently weakened by the high abundance of *D. marginatus* at the study site. This abundance may reduce the advantages to the settlers from joining conspecific adults, since large patches of the reef can be considered as covered and protected by adults during foraging and thus provide a better survivorship for the settlers (similar to the settlers of *D. aruanus* that settle near their adults). Moreover, individual specimens (males) that were observed nesting (Ben-Tzvi, unpublished data) suggest that, for this fish, due to their density at the study site, the harem is not essential for reproduction and, thus, finding a future mate should not be a factor influencing the settlers' decision.

While the habitat selection of *D. marginatus* seems to be random regarding conspecific adults, some consistency was observed in the choice of the same specific specimens of coral colonies (regardless of their species). This pattern may indicate that the fish select their microhabitat according to some (unknown) qualities of the coral colonies, as shown for *C. viridis* [22]. Even if the absence of settlers in *S. pistillata* is the result of predation, the observed consistency in settlement in other corals indicates that the distribution of *D. marginatus* settlers is not random. The high proportion of these colonies occupied by adult *D. marginatus* indicates that these fish do migrate at some (later) stage, as other pomacentrids do [19].

The contrasting findings of previous [16], [39] and our own studies could be related to such factors as the different conditions (e.g. lower food availability that makes the cost of aggregation higher, and lower predation that reduces the benefit from adult protection) to which the distinct populations have been exposed for many generations. However, we believe that this is not the case and that the different results may also be partly due to the differences in the experimental/observation set-ups of the different studies. Sweatman conducted his experiments in an artificial set-up. His Artificial Coral Units (ACU) were widely separated and juveniles did not have the option of settling to unoccupied coral heads near these ACUs (in order to reduce the cost of the adult aggression), whereas we worked on continuous reefs where such an option did exist. Moreover, the use of conspecific cues in habitat detection may be very important in sparsely distributed habitats, such as Sweatman's set-up with its ACUs spread far apart on sandy bottom [e.g. 15]. However, in areas with more densely packed habitats, it is possible for the settlers to use the same cues in order to find an area with a high quality habitat, and then settle nearby conspecifics. There may also be a spatial scale effect here: conspecific cues may be important over larger distances (10 s to 100 s of m) in order to home in on a suitable reef, while at smaller scales (meters and even less) settlers are already in the neighborhood of a suitable habitat and could perhaps choose appropriate settlement sites without relying on conspecific cues. Alternatively, the cues may not be strong enough to make all the recruiting fish concentrate only in a single coral head, especially if there are many potential settlement habitats available in the vicinity.

The present study emphasizes the need for further investigation of the important aspects of habitat selection during fish settlement and the effect of conspecific adult behavior in it, in the natural complex environment of the reef. In spite of the advantages of isolating one factor in the laboratory and studying its effects, the prediction of what might happen in the natural reef is not always straightforward. The “dilemma” of the settling *D. aruanus* that is described here reveals a new dimension in the complexity of habitat selection by the naïve fish upon their arrival at the coral reef. Although laboratory experiments are important to our understanding of fish habitat selection, further studies in the natural environment are essential in order to elucidate the actual patterns of settlement and habitat selection, which are critical for the replenishment and maintenance of coral reef fish populations.
